# Large-Diameter Burrows of the Triassic Ischigualasto Basin, NW Argentina: Paleoecological and Paleoenvironmental Implications

**DOI:** 10.1371/journal.pone.0050662

**Published:** 2012-12-05

**Authors:** Carina E. Colombi, Eliana Fernández, Brian S. Currie, Oscar A. Alcober, Ricardo Martínez, Gustavo Correa

**Affiliations:** 1 Consejo Nacional de Investigaciones Científicas y Técnicas, San Juan, Argentina; 2 Departamento de Paleontología de Vertebrados, Instituto y Museo de Ciencias Naturales, Universidad Nacional de San Juan, España 400 (Norte) San Juan, Argentina; 3 Department of Geology, Miami University, Oxford, Ohio, United States of America; Ludwig-Maximilians-Universität München, Germany

## Abstract

Large-diameter ichnofossils comprising three morphotypes have been identified in the Upper Triassic Ischigualasto and Los Colorados formations of northwestern Argentina. These burrows add to the global record of the early appearance of fossorial behavior during early Mesozoic time. Morphotypes 1 and 2 are characterized by a network of tunnels and shafts that can be assigned to tetrapod burrows given similarities with previously described forms. However, differences in diameter, overall morphology, and stratigraphic occurrence allow their independent classification. Morphotype 3 forms a complex network of straight branches that intersect at oblique angles. Their calcareous composition and surface morphology indicate these structures have a composite biogenic origin likely developed due to combined plant/animal interactions. The association of Morphotypes 1 and 2 with fluvial overbank lithologies deposited under an extremely seasonal arid climate confirms interpretations that the early appearance of burrowing behavior was employed by vertebrates in response to both temperature and moisture-stress associated with seasonally or perpetually dry Pangean paleoclimates. Comparisons of burrow morphology and biomechanical attributes of the abundant paleovertebrate fauna preserved in both formations permit interpretations regarding the possible burrow architects for Morphotypes 1 and 2. In the case of the Morphotype 1, the burrow constructor could be one of the small carnivorous cynodonts, *Ecteninion* or *Probelesodon*. Assigning an architect for Morphotype 2 is more problematic due to mismatches between the observed burrow morphology and the size of the known Los Colorados vertebrates.

## Introduction

Over the last two decades, tetrapod-burrow casts with diameters greater than 10 cm have been identified across broad paleolatitudinal gradients of the supercontinent Pangea. These structures have been recorded in South Africa, Antarctica, North America, Europe, and South America and are an indicator of a relatively common tetrapod behavior during Permo-Triassic time (e.g. [1]–[13]). The South African burrows are particularly important because of their internal preservation of small-sized therapsid fossils, interpreted as the remains of the burrow architect [1], [2]. Despite the lack of skeletal material preserved in other Permo-Triassic burrows, these findings have been used to hypothesize that the burrowers of contemporaneous large-diameter burrows (>10 cm) were also small therapsids (e.g. [4], [6], [12]).

In spite of several reports of burrows from South America (e.g. [11], [14]), the first burrows to be described in detail from this continent are derived from the Upper Triassic Ischigualasto Formation from the Ischigualasto-Villa Union Basin of Argentina [7]. Here, we build on this previous report of large-diameter burrows. Three different types of large diameter cylindrical structure are described in detail below. Two morphotypes are characterized by networks of tunnels and shafts that can be assigned to tetrapod burrows given similarities with previously described forms [4]–[6]. A third morphotype is interpreted as forming as a result of interaction between burrowing invertebrate (or vertebrate) and coeval root systems.

Many researchers interpret the global early Mesozoic appearance of the tetrapod burrows as a behavioral adaptation evolved by terrestrial vertebrates as protection against extreme climatic conditions created during the tectonic assembly of, and by the paleolatitudinal setting of, the supercontinent Pangea [5], [6], [9], [15]. Low to mid-latitude Pangean climates are interpreted as having been highly seasonal in nature and characterized by long dry periods and a short wet season (e.g. [16]–[20]). The burrows of the Ischigualasto-Villa Union Basin are exclusively associated with depositional facies that have been interpreted as being deposited under similar climatic conditions [7], [21]. The ichnofossils appear together with abundant vertebrates fossils in floodplain facies of high-sinuosity rivers and are associated with mature calcisols, confirming the link between early burrowers and extreme climatic conditions.

### Geological Setting

**Figure 1:**
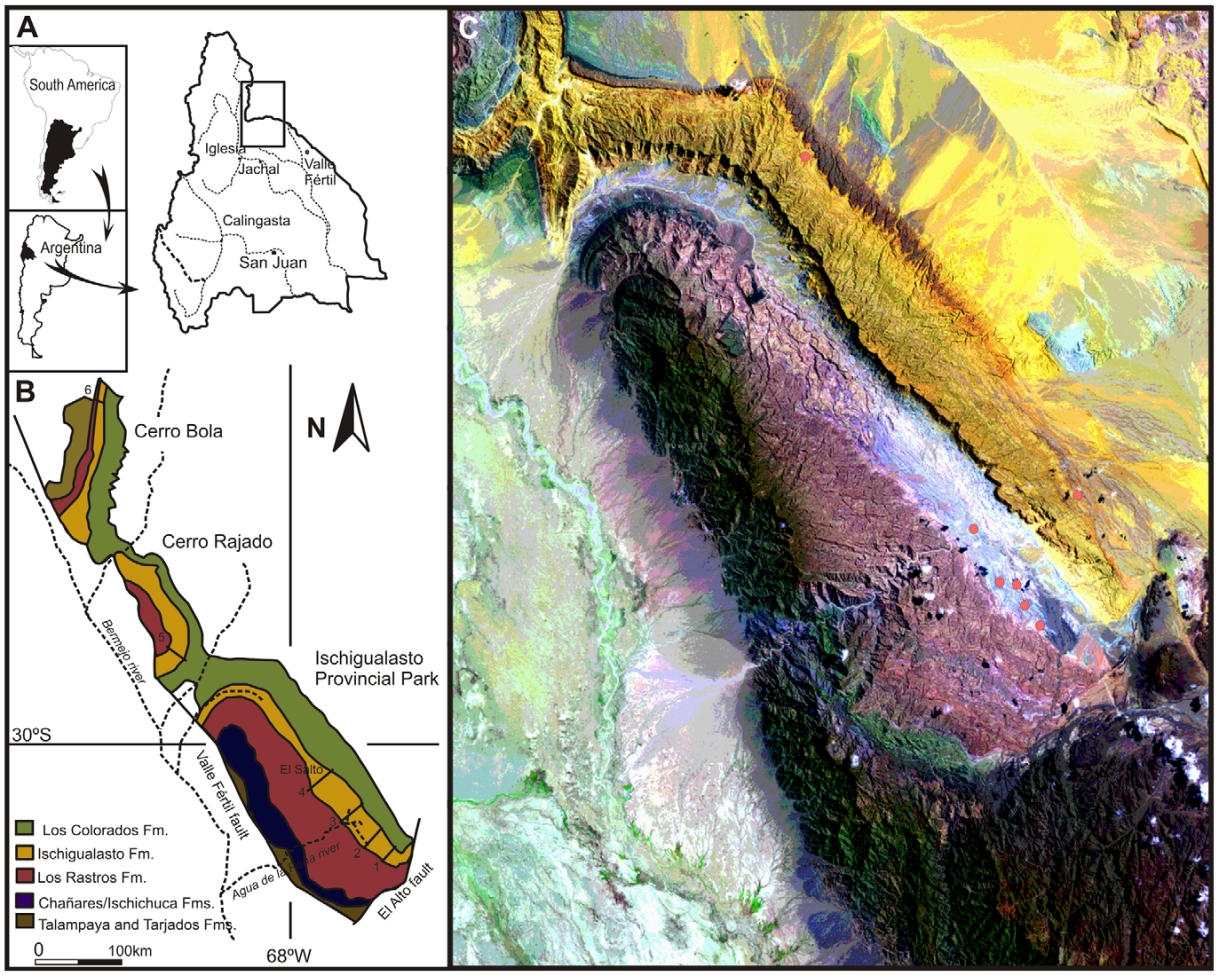
Study area location maps. (A) Location of the Triassic Ischigualasto-Villa Unión Basin in northwestern Argentina showing location of geologic map in Figure 1B (reproduced from Figure 1 of Colombi et al. [7]). (B) Geological map of the Triassic Ischigualasto-Villa Unión Basin showing position of satellite image shown in Figure 1C. (C) False color satellite image of the southern part of the basin. Red dots mark the locations of large diameter burrows identified in the Ischigualasto and Los Colorados Formations.

**Figure 2:**
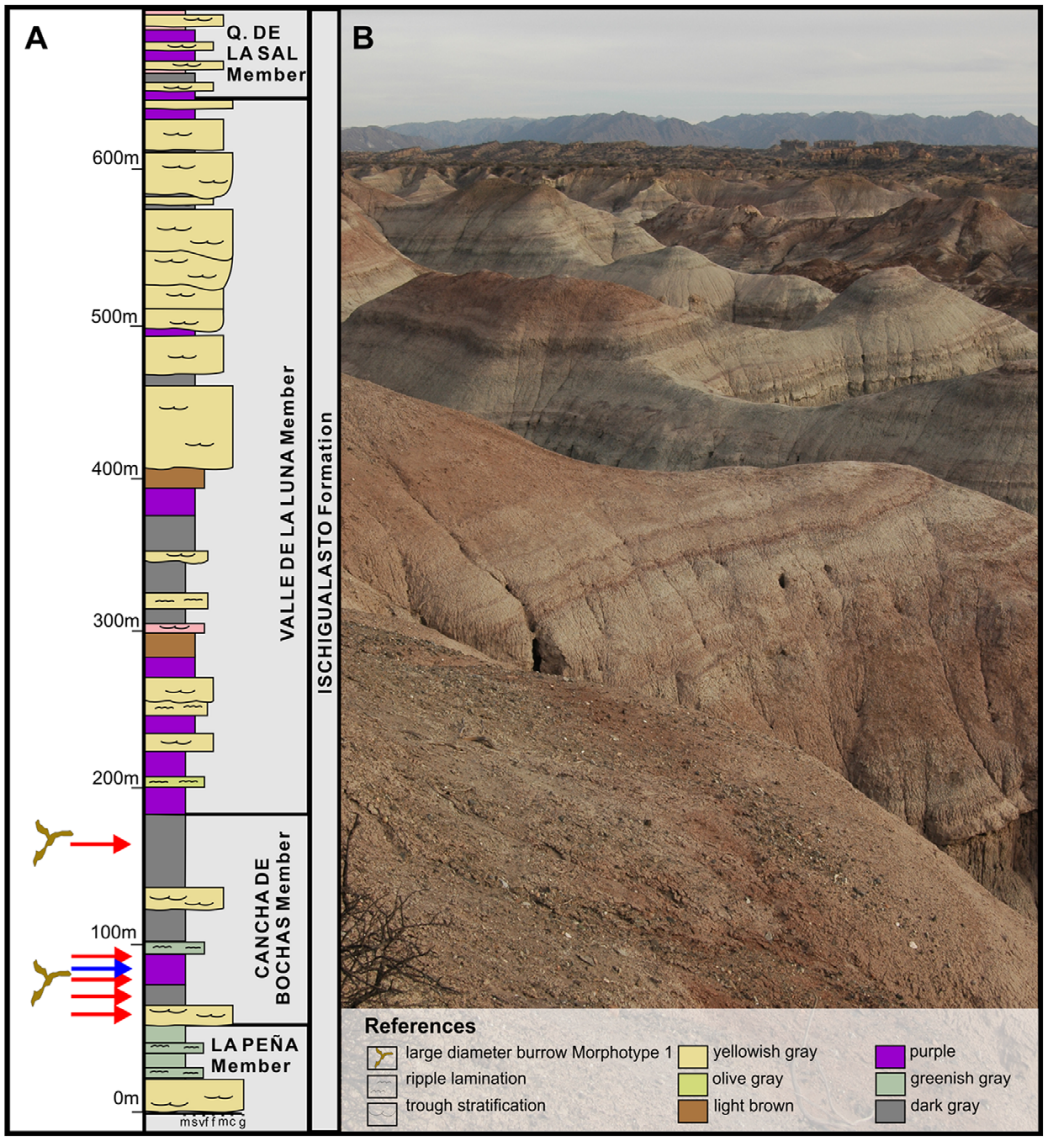
Stratigraphic positions of morphotypes 1 and 3 in Ischigualasto Formation. (A) Generalized stratigraphic section of the Ischigualasto Formation. Red arrows indicate the stratigraphic position of the Morphotype 1 burrow casts identified in the Cancha de Bochas Member, while blue arrows indicate the Morphotype 3 burrow casts. (B) Photograph of typical overbank lithologies in the Cancha de Bochas Member that host the observed large-diameter burrows.

The Ischigualasto-Villa Union Basin is one of a series of early Mesozoic continental-rift basins that formed along the southwestern margin of Pangea [22]. The fossil burrows reported here were identified in the Upper Triassic Ischigualasto and Los Colorados formations, in San Juan and La Rioja provinces, northwestern Argentina (Figure 1). In the study area the Ischigualasto Formation is comprised of ∼350–700 m of fluvial channel sandstones and conglomerates, and intercalated levee, crevasse splay, and floodplain sandstones and mudstones (Figure 2) deposited by low and high sinuosity fluvial systems. The formation also contains numerous layers of altered volcanic ash [23], [24]. Two of these layers, one located near the bottom and the other near the top of the Ischigualasto Formation, have been dated by radiometric techniques and indicate a Carnian–Norian depositional age of between ∼231 and 225 Ma [25], [26], based on the time scale of Walker and Geissman [27]. The Ischigualasto Formation contains four stratigraphic members that are differentiated on the basis of lithological content, sedimentological architecture and paleosol morphology [24]. In ascending orde these include the La Peña, Cancha de Bochas, Valle de la Luna and Quebrada de la Sal members (Figure 2). The taphonomic attributes of the paleoflora and paleovertebrates collected from the Ischigualasto Formation differs between the stratigraphic members indicating that the sedimentological/paleopedological criteria used to define the members likely developed due to changes in paleoclimatic and tectosedimentary conditions during the time of deposition [28], [29].

The large-diameter burrows of the Ischigualasto Formation described in this report come exclusively from the Cancha de Bochas Member, which is characterized by high-sinuosity channel facies interlayered with well-developed calcic paleosols that host most of the Ischigualasto paleovertebrates. This interval has been interpreted as being deposited during a period of relatively low sedimentation rates under an extremely seasonal arid climate [21], [24], [27], [28].

**Figure 3:**
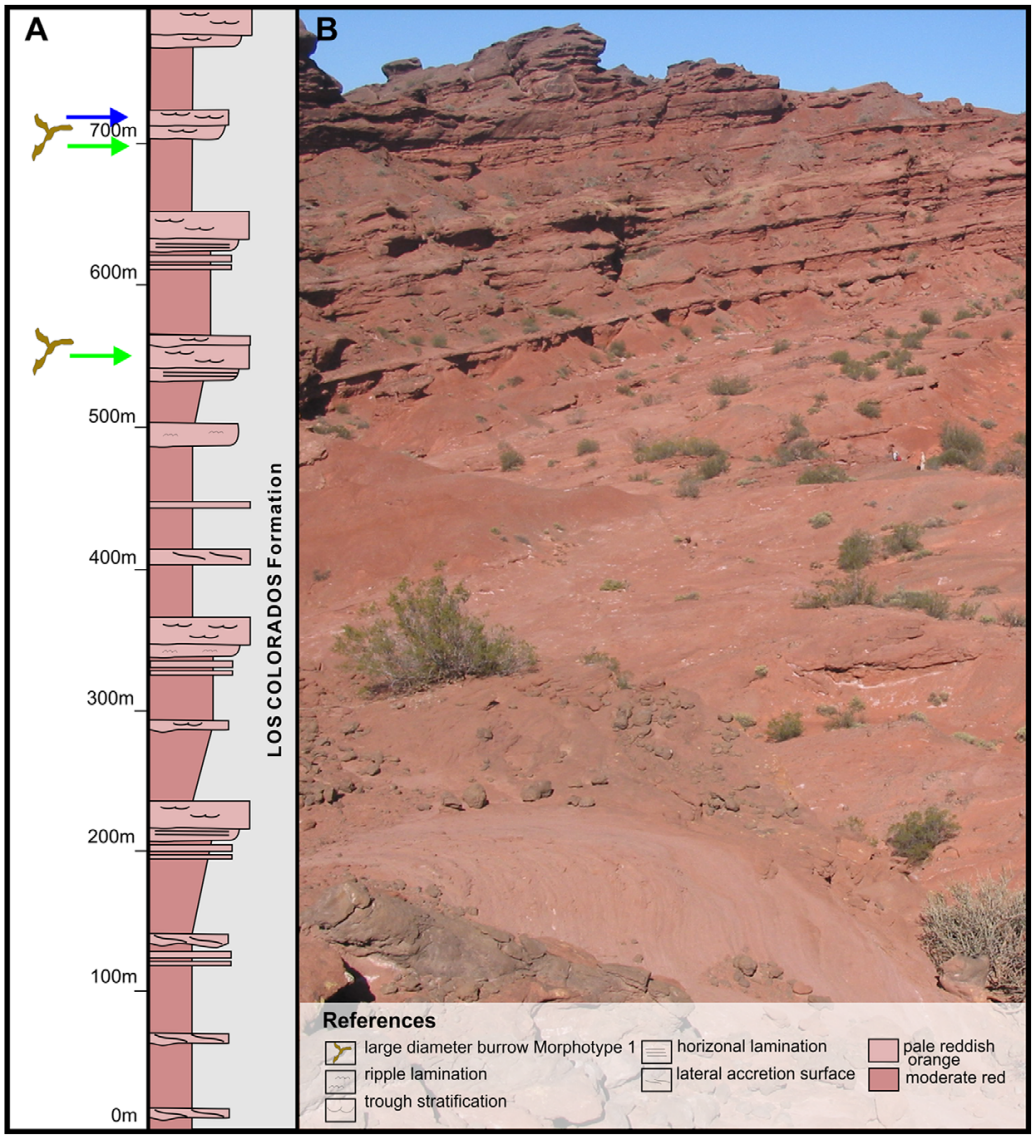
Stratigraphic positions of morphotypes 2 and 3 in Los Colorados Formation. (A) Generalized stratigraphic section of the Los Colorados Formation. Green arrows indicate the stratigraphic position of Morphotype 2 burrow casts identified in the upper ∼150 meters of the succession, while blue arrows indicate Morphotype 3. (B) Photograph of typical fluvial channel/overbank lithologies in the upper Los Colorados Formation.

The Los Colorados Formation conformably overlies the Ischigualasto Formation and ranges in thickness from approximately 500 m to 700 m [30], [31]. The unit is comprised of fluvial-channel sandstones and overbank sandstones and mudstones (Figure 3) [30]. The depositional age of the Los Colorados Formation has not been radiometrically defined. However its stratigraphic continuity with the underlying Ischigualasto Formation allows the assignment of a Norian age. Additionally, a recent magnetostratigraphic study has reported a Norian age for the entire Los Colorados Formation [32].

The large-diameter burrows observed in the Los Colorados Formation are concentrated in the upper 150 m of the unit. This interval is characterized by high-sinuosity channel deposits and associated overbank lithologies that contain mature calcic paleosols. The interval also contains most of the vertebrate fossils preserved in the Los Colorados Formation [31]. Similar to the burrowed intervals of the Ischigualasto Formation, the sedimentological, paleopedological and taphonomical characteristics of the upper Los Colorados Formation indicate low sedimentation rates and a highly seasonal xeric climatic during the time of deposition.

### Paleontological Setting

The Upper Triassic of the Ischigualasto Basin is well known for its rich paleofaunal record which covers different habitats and sizes, including small (<25 kg), medium (25–200 kg) and large (>200 kg) tetrapods.

The Carnian–Norian Ischigualasto Formation presents one of the worldwide most diverse Upper Triassic faunal records. The formation contains several of the best-known earliest dinosaurs, as well as archosauromorphs, crurotarsan archosaurs, therapsids and amphibians. Nevertheless, the diversity and abundance of fossils is variable, both laterally within individual stratigraphic intervals, and vertically through the formation. Martínez et al. [27] divided the formation into three abundance biozones limited by local extinctions. The burrow casts from the Ischigualasto Formation are concentrated in the *Scaphonyx*-*Exaeretodon*-*Herrerasaurus* biozone (S-E-H biozone), which is oldest stratigraphically, and contains the highest diversity and abundance of fossils within the formation.

Therapsids are one of the most abundant and diverse groups of vertebrates of the *S*-*E*-*H* biozone. Among the therapsids, the cynodonts are the most diverse, as represented by the highly abundant, medium-sized, herbivorous *Exaeretodon* and *Ischignathus* [33], [34], the small-sized, faunivorous *Ecteninion*, *Probelesodon*, and a juvenile specimen of cf. *Probainognathus* [35]–[37].

The other group of therapsids contains the large-sized, herbivorous dicynodonts *Ischigualastia* [38] and *Jachaleria* [27], [39]. The other group of abundant paleovertebrates is the mid-sized, herbivorous archosauromorph *Scaphonyx* [40], which represents ∼60% of the fossils in this interval [27]. The biozone also includes some of the best-known early dinosaurs, such as the small-sized *Pisanosaurus*, *Eoraptor*, *Panphagia*, *Eodromaeus*, and *Chromogisaurus* [27], [41]–[44], and the mid-sized herrerasaurids *Herrerasaurus* [45], [46] and *Sanjuansaurus* [47].

The *S*-*E*-*H* biozone also contains a very diverse but less abundant group of vertebrates, the crurotarsan archosaurs. This group includes the medium-sized sphenosuchian *Trialestes* [48], the ornithosuchid *Venaticosuchus* [49], the armored aetosaur *Aetosauroides* [50], the poposaurid *Sillosuchus* [51] and the large-sized “rauisuchid” *Saurosuchus* [52]. In addition this biozone includes the archosauriforms *Proterochampsa* [53] and *Chanaresuchus ischigualastensis* [54].

The upper Los Colorados Formation preserves a highly diverse paleovertebrate fauna characterized by its unusual combination of abundant dinosaurs and a high diversity of crurotarsan archosaurs, derived therapsids, and primitive chelonians. The most substantial change between the dinosaur fauna from the Ischigualasto Formation and that of the upper Los Colorados Formation is the increasing body-size from the older to the younger unit, for both carnivores and herbivores [27].

The most abundant vertebrate in the Los Colorados Formation is the large-sized basal sauropodomorph dinosaur *Riojasaurus* [55], which represents 40% of observed specimens. Other dinosaurs present are basal sauropodomorphs including the large-sized *Coloradisaurus* [56] and *Lessemsaurus* [57], [58], as well as the uncommon, large-sized theropod *Zupaysaurus* [59]. Crurotarsan archosaurs are highly diverse in the Los Colorados Formation and include the medium-sized, armored aetosaur *Neoaetosauroides* [60], the small-sized sphenosuchid *Pseudohesperosuchus* [49] and the protosuchid *Hemiprotosuchus* [49], the medium-sized ornithosuchid *Riojasuchus* [49], and the large-sized rauisuchid *Fasolasuchus* [61], [62].

Despite the relative abundance and taxonomic diversity of tetrapods from the upper Los Colorados Formation, therapsids are relatively uncommon and are represented by the tritheledontid *Chaliminia* [63], [64] and a probable tritylodontid [49]. A final minor component of the fauna of the Los Colorados Formation is the small-sized, chelonian *Palaeochersis* [65].

## Methods

The Instituto and Museo de Ciencias Naturales has all the necessary permits needed to explore the Ischigualasto Provincial Park area, and unearth and study the fossil materials described in this paper. This work complies with all relevant regulations.

**Figure 4:**
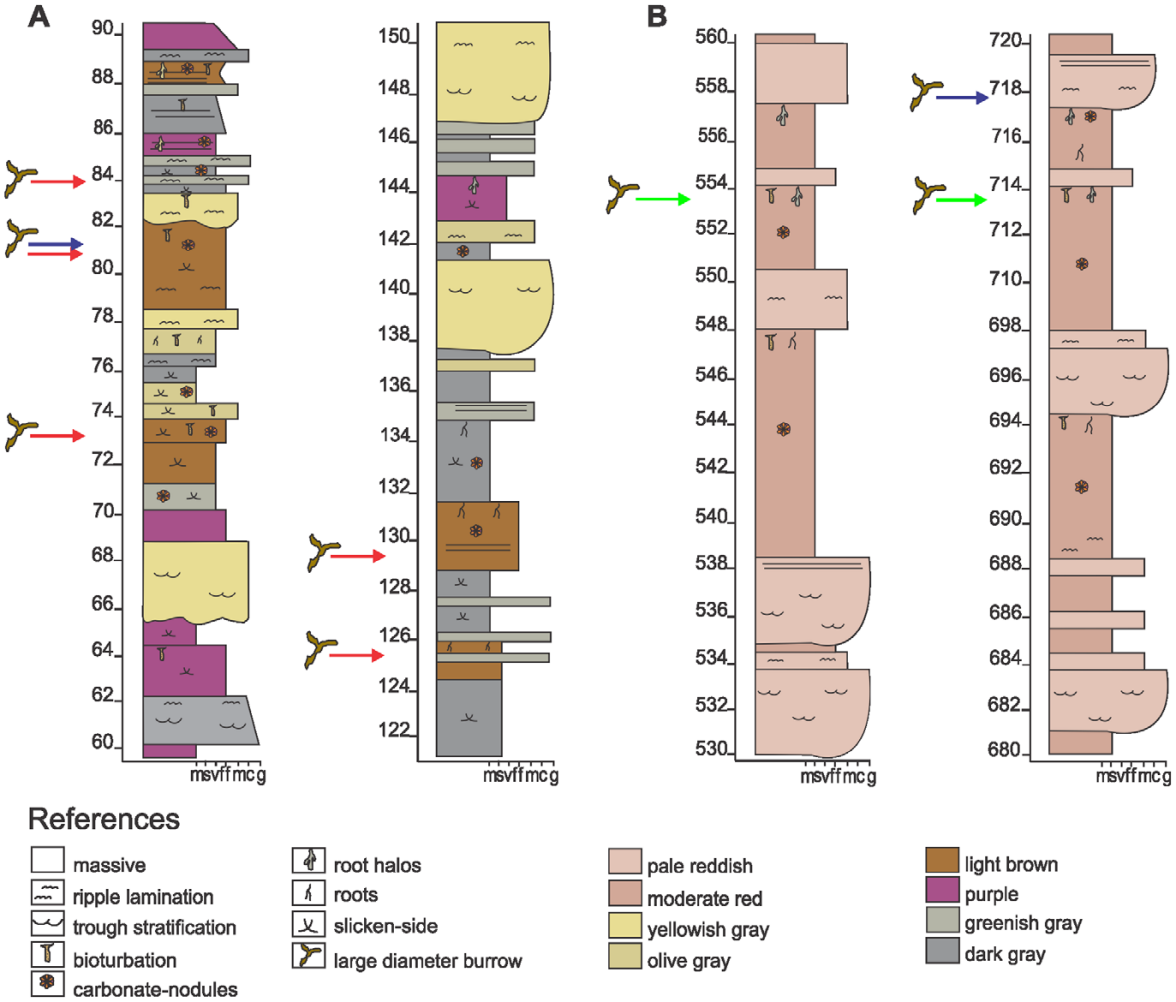
Detailed statigraphic columns showing the sedimentology of the study interval. (A) Detailed stratigraphic section of Ischigualasto Formation, red arrows indicated the stratigraphic position of Morphotype 1 burrows, while blue arrow indicates Morphotype 3. (B) Detailed stratigraphic section of Los Colorados Formation, green arrows indicate the stratigraphic position of Morphotype 2 burrows and blue arrow indicates the position of Morphotype 3 burrows.

The large-diameter (>10 cm) structures studied here are contained mainly within overbank facies of the Upper Triassic succession. Nine different stratigraphic horizons were studied in detail (Figures 2, 3, 4), including six in the Canchas de Bochas Member of the Ischigualasto Formation, and three in the upper part of the Los Colorados Formation. These fossils were studied in the field and have not been collected. Their stratigraphic positions are specified in Table 1 using meters from the base of the Ischigualasto Formation or Los Colorados Formation as relevant. Geographic localities are specified in Table 1 using Universal Transverse Mercator Coordinates.

**Table 1:** Stratigraphic position and geographic localities of burrows under study.

**Burrow cast** **morphotype**	**Stratigraphic** **level**	**Coordenates** **(UTM)**
Morphotype 1	73m Ischigualasto Fm.	19J 0608102/6665462
	81m Ischigualasto Fm.	19J 0608104/6665472
	84m Ischigualasto Fm.	19J 0605726/6667537
	126m Ischigualasto Fm.	19J 0605975/6666991
	130m Ischigualasto Fm.	19J 0605112/6669072
Morphotype 2	553m Los Colorados Fm.	19J 0611681/6669315
	713m Los Colorados Fm.	19J 0591039/6694788
Morphotype 3	83m Ischigualasto Fm.	19J 0607665/6665615
	717m Los Colorados Fm.	19J 0591040/6694785

UTM, Universal Transverse Mercator Coordinates.

A characterization of these structures has been made primarily on the basis of ichnotaxabases that account for the architectural and surficial morphology of the burrow casts, complexity and tortuosity indices, and the fill type [6], [66]. Evaluation of architectural morphologies includes general dimensions, cross-section geometry, spatial orientation, type of branching, and burrow-element interconnectedness. Surficial morphology refers to both large and diminutive structures on the surfaces of walls. Descriptions and interpretations regarding the origin of these burrows are listed below.

## Results

### Morphotype 1

This morphotype from the Ischigualasto Formation was preliminarily described and interpreted as a tetrapod burrow cast by Colombi et al. [7]. These burrows appear in the Cancha de Bochas Member, where five individual burrowed horizons have been identified (Figures 1, 2, 4). The burrows are preserved in two types of overbank facies of a high-sinuosity fluvial system (Figures 2, 4). Three of the burrow horizons are in levee facies that consist of reddish-brown colored, structureless to ripple-cross laminated, fine- to medium-grained, muddy-sandstone and sandy-mudstone. The burrows are also developed in sandy-crevasse splay facies, characterized by structureless or ripple-cross laminated greenish-gray muddy sandstone. All facies are highly bioturbated by invertebrate burrows and overprinted by pedogenic structures including hydroximorphic mottles, root halos and traces, slicken-sided peds and abundant pedogenic carbonate nodules and rhizoconcretions [21], [24]. The Cancha de Bochas Member paleosols associated with the burrowed intervals have been interpreted as calcic vertisols, calcisols and argillic calcisols [21], [24].

**Figure 5:**
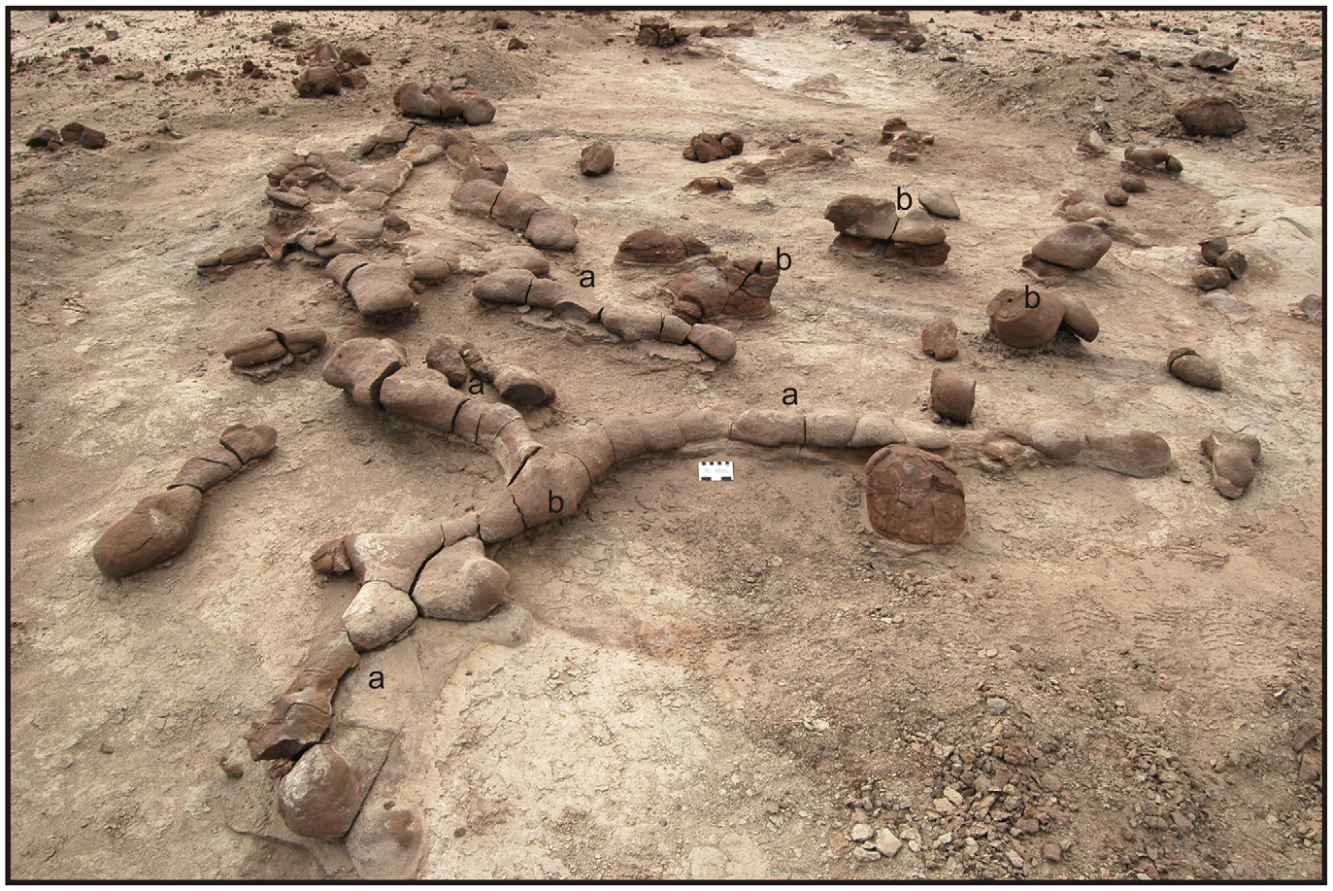
Morphotype 1. Photograph of a general view of a typical Morphotype 1 burrow complex (modified from Figure 3.1 of Colombi et al. [7]). Note the tunnels with medial and terminal chambers (A) and the vertical shaft intersecting one of the primary tunnels (B).

**Figure 6:**
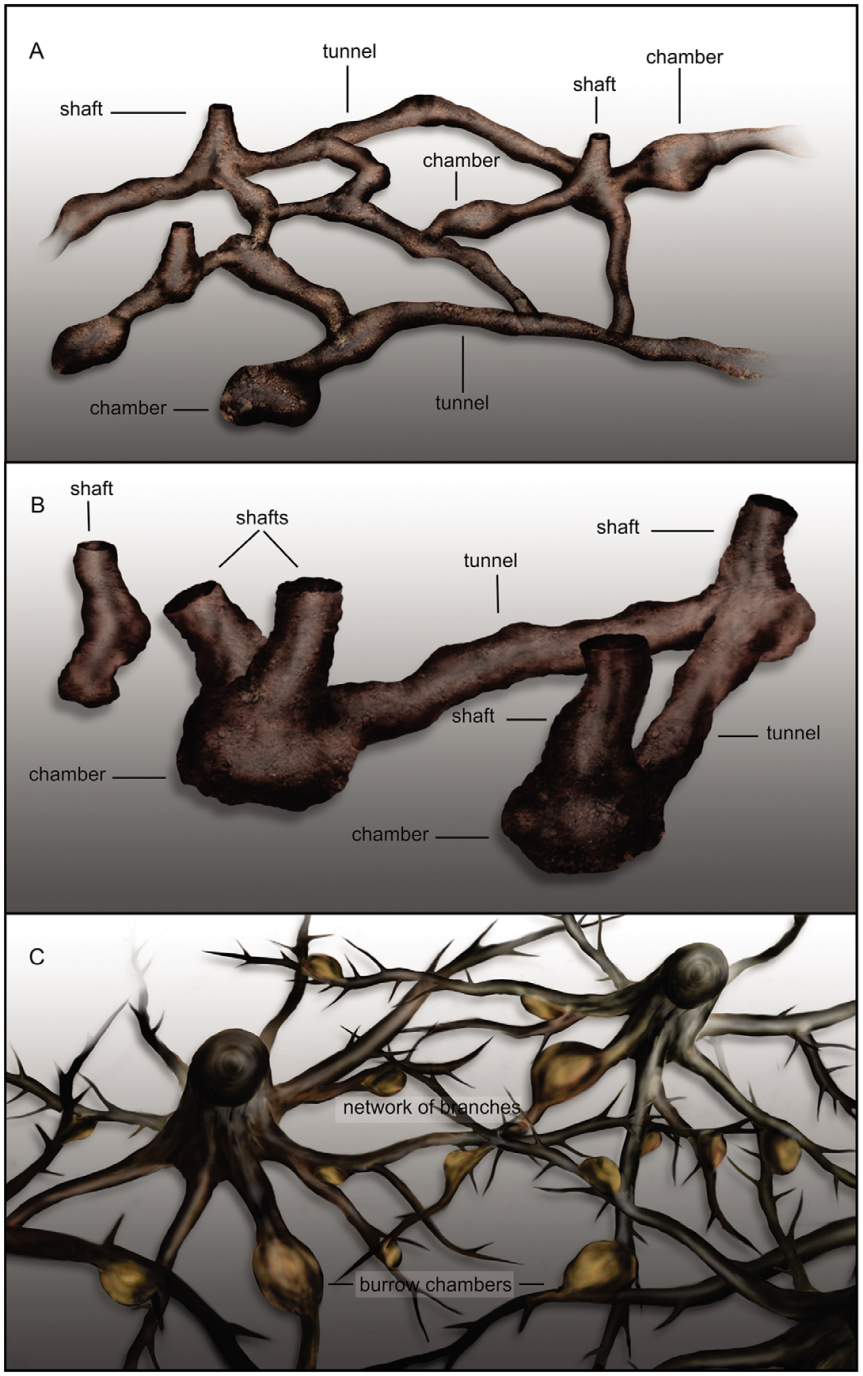
Schematic diagrams explaining the nature of the large diameter burrow morphotypes. (A) Morphotype 1. (B) Morphotype 2. (C) Morphotype 3.

**Figure 7:**
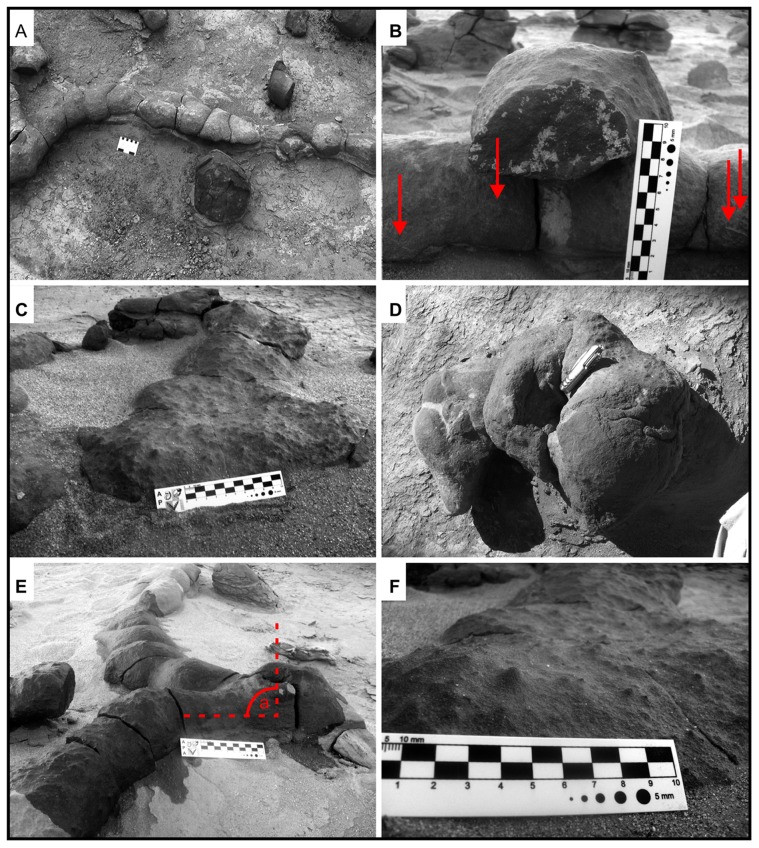
Main ichnotaxabases of Morphotype 1 burrow complexes. (A) Straight to slightly sinuous tunnels that reach 1 meter in length. (B) Cross section of a tunnel displaying the roughly elliptical geometry and the longitudinal medial groove along the base of the burrow (modified from Figure 3.3 of Colombi et al. [7]). Note the longitudinal ridges indicated by red arrows. (C) Terminal chamber with an average diameter of 25 centimeters. Note the gradual increase of the diameter from the tunnel to the chamber (modified from Figure 3.4 of Colombi et al. [7]). (D) Vertical shafts in the burrow complex. Note the central pit produced by differential cementation of the fill material. (E) Relationship between shaft and the tunnels. Note the perpendicular angle between tunnels and shaft (a) (modified from Figure 3.5 of Colombi et al. [7]). (F) Surficial morphology along the sides and tops of the burrows displaying the characteristic granular texture produced by bioturbation.

The individual large-diameter burrow systems consist of horizontal to subhorizontal tunnels and short vertical shafts that cover areas of up to 2 m^2^, (Figures 5, 6). Tunnels are straight to slightly undulatory, and reach maximum lengths of 1 m (Figure 7a). The diameters of the burrows average 10 cm (with maximum diameters of ∼15 cm), and display uniform, roughly elliptical cross-sectional geometries. In some segments of the tunnels, the floor bears a longitudinal medial groove that forms a shallow U-shape when viewed in transverse cross section (Figure 7b). The tunnels contain intermediate and terminal enlargements, interpreted as chambers, with average diameters of ∼25 cm (Figure 7c). The greater diameters of the chambers are attained by a gradual increase in tunnel diameter. Vertical shafts in burrow complexes are less than 20 cm long, although their original length may have been reduced due to compaction or erosional truncation (Figure 7d). Shafts, which likely represent burrow entrances, are commonly located at tunnel intersections or at the beginning of tunnels. The shafts are connected perpendicularly to horizontal/subhorizontal tunnels (Figure 7e). The branching angle of tunnel segments is ∼90°, forming a T-shape branching (Figure 5, 7e). The tortuosity index of the burrows (T) is 1.3, indicating the simple geometry of the branching. The complexity index of the Ischigualasto burrows is difficult to define because of incomplete burrow-cast preservation.

The fill of the studied ichnofossils consists of brown, medium-grained, carbonate cemented sandstone. The boundaries between the burrow fill and hosting facies are very well defined. Approximately half of the observed shaft molds contain a central pit produced by differential cementation of the fill material (Figure 7d). The surficial morphology along the sides and tops of the burrows consists of poorly defined longitudinal ridges, 2–3 mm wide, which likely represent scratch marks [5] (Figure 7b). All surfaces exhibit a granular texture created by bioturbation (Figure 7f).

### Morphotype 2

These large-diameter structures appear in the upper 150 m of the Los Colorados Formation (Figures 3, 4). They occur as cylindrical structures developed in red, structureless to weakly-laminated mudstones interpreted as overbank deposits of a meandering fluvial system. Similar to the burrowed intervals in the Ischigualasto Formation, these facies are overprinted by calcic paleosols displaying calcic nodules, scarce hydroximorphic mottles, and argillic-cutans. Following interpretations of similar Ischigualasto Formation paleosols, these paleosols are classified as argillic-calcisols [21].

**Figure 8:**
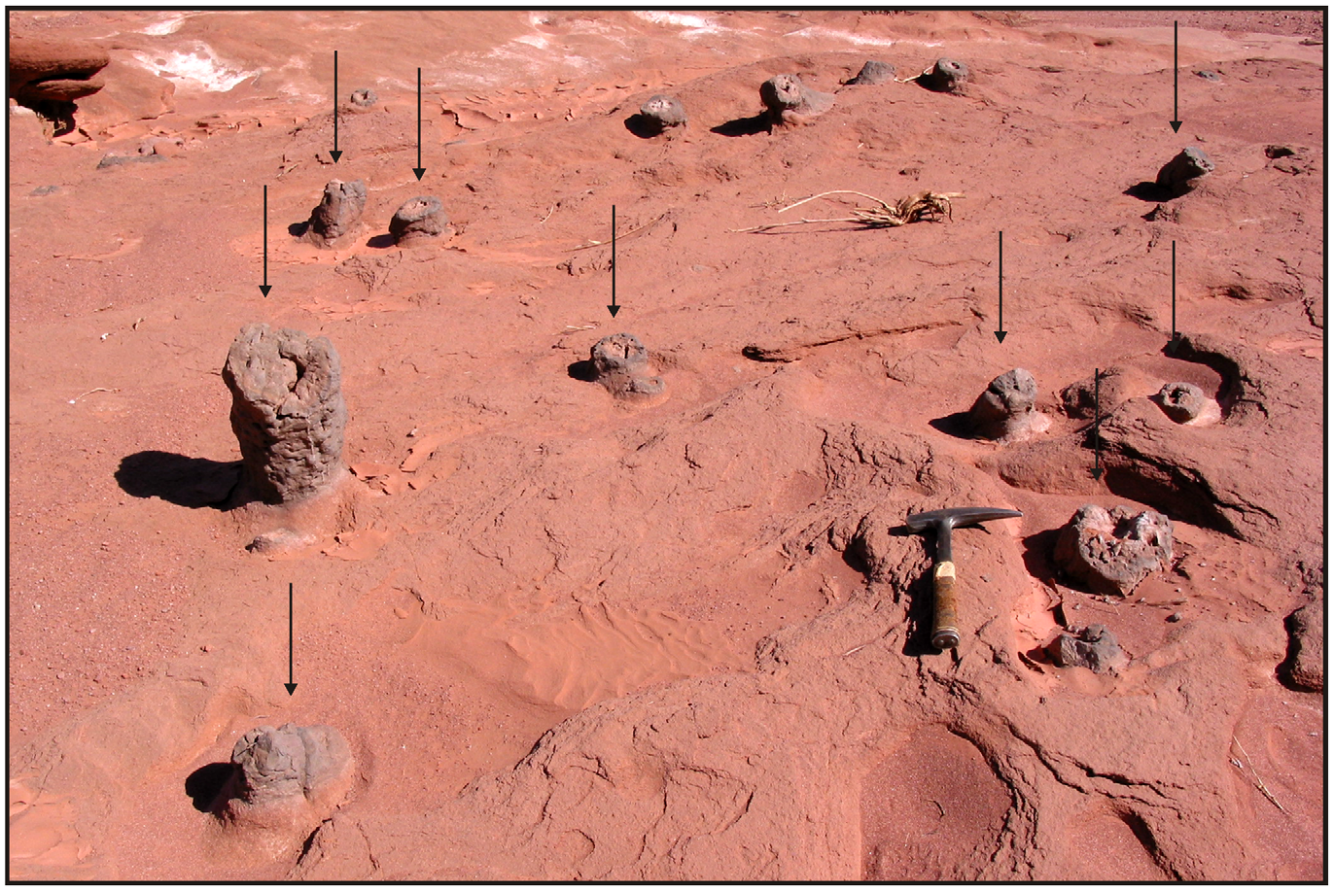
Morphotype 2. Photograph of a general view of Los Colorados burrow complexes. Note the numerous exhumed vertical shafts outcropping across the landscape (arrows).

**Figure 9:**
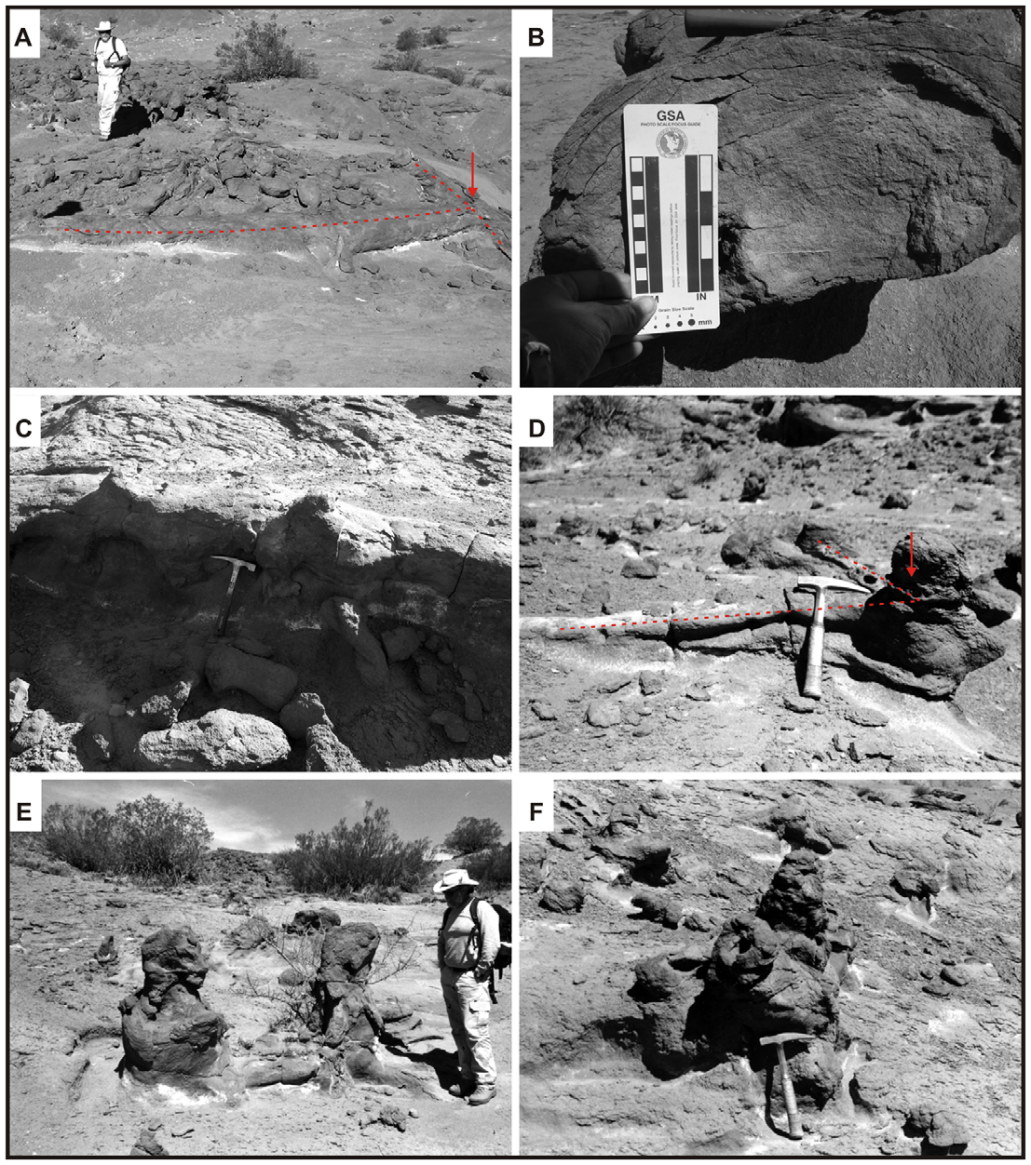
Main ichnotaxabases of Morphotype 2 burrow complexes. (A) Straight tunnels intersecting at right angles (arrow). (B) Elliptical cross section showing a near maximum burrow diameter of ∼45 centimeters. Note the flattened tunnel base. (C) Wavy tunnel base viewed in a longitudinal section. (D) Slight enlargement at the intersection between a tunnels and a vertical shaft. Note the 90° angle of the tunnels intersection (arrow). (E) Vertical shafts that characterize the burrow casts preserved in Los Colorados Formation. The shafts extend over a meter above lower tunnels. (F) Common manifestation of shafts in pairs with similar morphological characteristics. See the central pits of the shafts produced by differential cementation of the fill material.

Burrow Morphotype 2 consists of a simple elongated network displaying one or two horizontal or subhorizontal tunnel-like structures and several vertical cylindrical structures (Figures 6, 8, 9). The complexes cover areas of 4–8 m^2^ (Figure 8). Their geometries are notably simpler than the burrow complexes preserved in the Ischigualasto Formation, but have a higher density distribution within individual stratigraphic intervals. In some instances individual burrows are superimposed on older forms (Figure 8).

Tunnels in this morphotype are straight or slightly curved to one side (Figure 9a). They are elliptical in cross section, with horizontal diameters averaging 35 cm (with a maximum diameter of ∼50 cm) and vertical diameters averaging 20 (maximum diameter 30 cm) (Figure 9b). Tunnel floors are flattened in cross-section and display a wavy profile in longitudinal transects (Figure 9c). In horizontal segments, enlargements occur at major burrow intersections or where vertical cylinders connect with two or more horizontal segments (Figure 9d). The branching angle of Morphotype 2 tunnel-like segments is ∼90°, and produces a “T-shaped” branching pattern (Figure 9d). The tortuosity index of observed horizontal segments is 1.

Vertical shafts of Morphotype 2 are up to one meter in length, although original vertical dimensions may have been compressed due to sediment compaction or erosional truncation (Figure 9e). Vertical structures are commonly observed in lateral pairs displaying similar dimensions and morphological characteristics (Figure 9f and 9e). They intersect both individual and multiple horizontal-burrow segments (Figure 9d).

**Figure 10:**
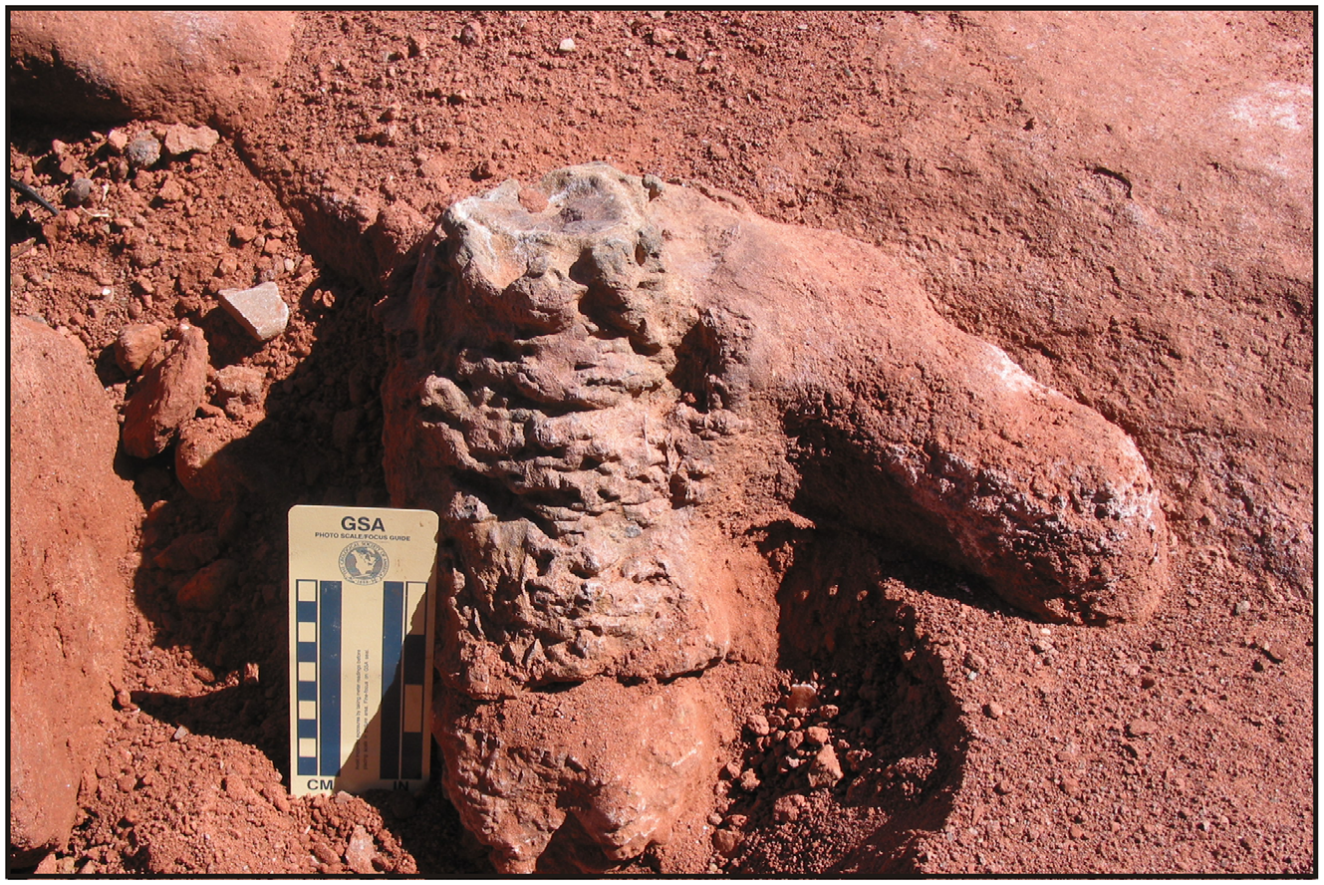
Wrinkled surface texture of one of the Morphotype 2 burrow casts. The wrinkled texture could be produced as a consequence of likely vertical flattening of moist/saturated host sediment.

The surface morphology of Morphotype 2 is for the most part smooth. However, the bases of some vertical structures display irregular vertically flattened surfaces (Figure 10). The burrow fill material consists of reddish-brown, medium-grained sandstone, cemented by carbonate. The margins between the burrow cast and the hosted rocks are very well defined due to the calcite cement and coarser-grained nature of the fill relative to the hosting mudstone. Almost all vertical structures contain a central pit produced by the differential cementation of the fill material (Figure 9f).

The overall morphology of Morphotype 2 most closely resembles a network of tunnels and vertical shafts generated by burrowing tetrapods (cf. [2], [5]–[7]). The enlargements observed in the horizontal structures are interpreted as terminal and medial chambers, which preserves rugosity that resemble recent burrow development in moist or water-saturated sediments.

### Morphotype 3

**Figure 11:**
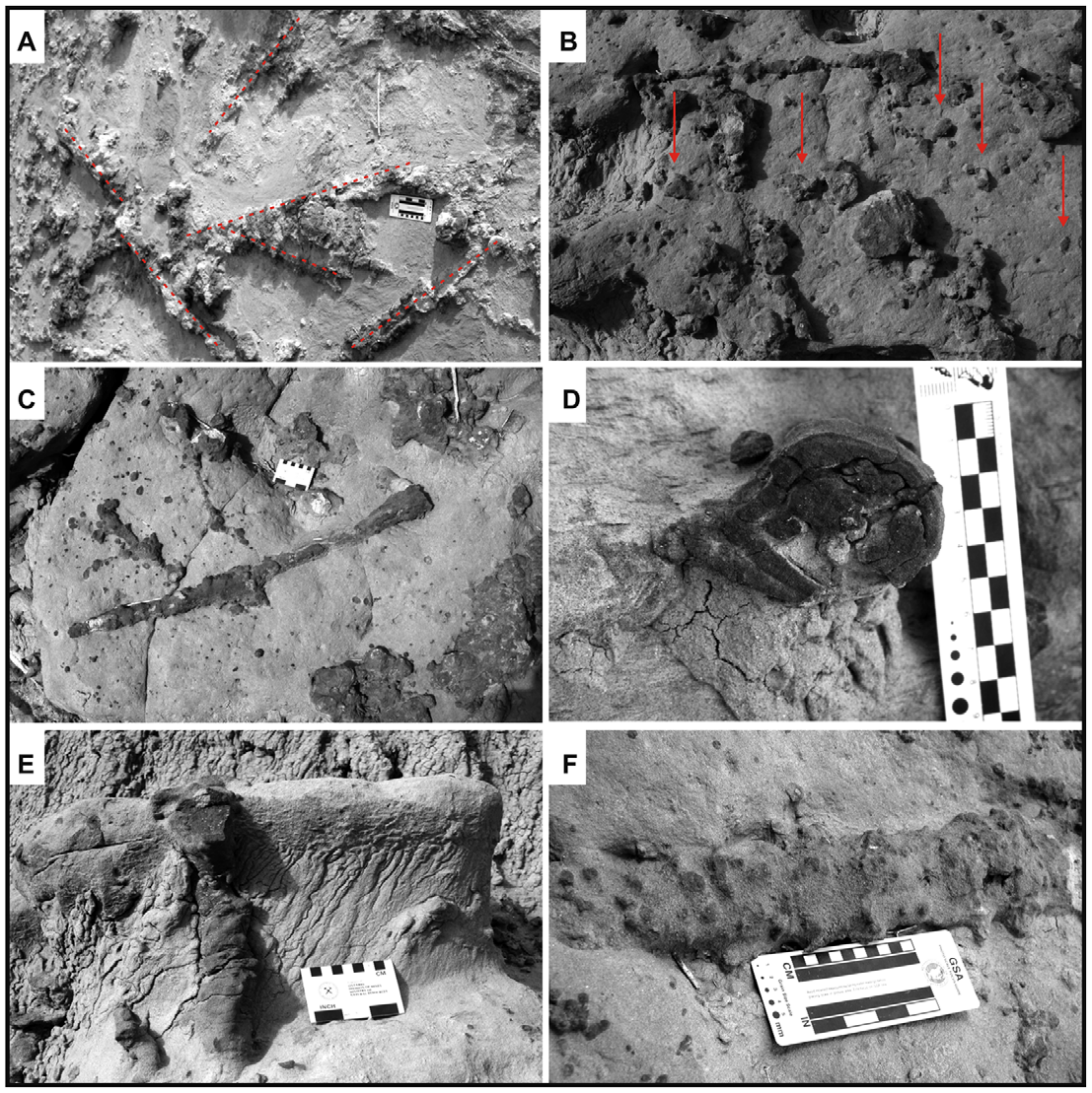
Main ichnotaxabases of Morphotype 3. (A) Complex network of straight branches cross-cutting at oblique angles (40°). Note the amalgamated associated calcium carbonate nodules (arrows). (B) Example of elliptical/flattened cross-sectional morphotype geometry. (C) Irregular surface of the rhizoconcretions, characterized by millimeter knobs and hummocks. (D) Horizontal branches that reach up to 3 meters in length. (E) Irregular diameter, shape, and surface morphology. (F) Vertical axes of more than 50 cm in length.

This morphotype appears in both Ischigualasto and Los Colorados formations in crevasse-splay deposits characterized by greenish-gray or red ripple-laminated sandy mudstone (Figures 2, 3, 4). In plan view, Morphotype 3 forms a complex network of straight branches (tortuosity index of 1) that intersect at oblique angles of about 40° (Figures 6, 11a). The diameter and shape of Morphotype 3 is highly irregular within and between individual structures (Figure 11b, c). The branches are >2.5 m in length and have elliptical/flattened-elliptical cross-sectional geometries (Figure 11d) with average diameters of ∼7 cm. Scarce vertical cylindrical structures with diameters up to 10 cm and more than 50 cm in length (Figure 11e) occur in close combination with these networks of horizontal branching.

Compositionally, Morphotype 3 structures are made entirely of micritic calcite containing isolated grains of sand or mud incorporated from hosting lithologies. Surfaces display numerous knobs and hummocks with a diameter of around 1 mm that result in an irregular texture (Figure 11f).

The morphotype is associated with overlapping amalgamated micritic sandy masses that reach 40 cm in diameter (Figure 11a, b).

## Discussion

The large diameter burrows of the Ischigualasto-Villa Union Basin add to the global record of the early appearance of fossorial behavior during early Mesozoic time.

Morphotypes 1 and 2 described above have important implications concerning the paleobiogeographical distribution of large non-marine burrowing organisms, as well as factors controlling the evolutionary development of burrowing behavior during the Late Triassic. The first workers who investigated large diameter burrows suggested that they had a latitudinally-controlled distribution [1], [2], [4], as is the case for modern burrowers, which are more prevalent at higher latitudes [67]–[69]. However, later reports documented diverse Permian-Triassic vertebrate ichnofossil assemblages from low- to high-paleolatitude locations, indicating no pronounced paleolatitudinal variation in this behavior during Permo-Triassic time (e.g. [1]–[3], [5]–[7], [9]–[12]). In the absence of a latitudinal control on the distribution of Triassic burrowing vertebrates, it has been postulated that burrowing may have been employed by early Mesozoic organisms at all paleolatitudes in order to counter the global extreme seasonal climate (e.g. [4], [6], [8], [70], [71]). Although initially developed to combat seasonal temperature fluctuations and water stress associated with seasonally dry climate regimes at low to mid-latitudes, burrowing behavior may have also allowed organisms to live at high latitudes by circumventing seasonal temperature fluctuations and perhaps serving as a refuge during winter dormancy [5]–[7], [72], [73].

Even though digging burrows may also be a response to increasing predatory pressure [5], [13], the Upper Triassic of the Ischigualasto-Villa Union Basin was deposited under a seasonal climate as evidenced by paleopedological [21], taphonomic [27], [28] and sedimentological studies [23], [24], [74], [75], indicating that climate had a strong influence on the appearance of burrows in this portion of Pangea. Moreover, the studied burrows appear to exclusively occur in isolated horizons of the Cancha de Bochas Member of the Ischigualasto Formation and the upper portion of the Los Colorados Formation, where in both cases a dry and highly seasonal climate is clearly evident (i.e., calcic soils, desiccation cracks, abundance of paleovertebrates, shrubby plants restricted to temporary rivers, etc.). These conditions are in agreement with other authors who have hypothesized that burrowing behavior was employed by vertebrates in response to both temperature and moisture-stress associated with seasonally or perpetually dry climates. Burrowing as an adaptive mechanism to seasonal droughts was first utilized by lungfish during the Devonian [76], and this strategy may have also been employed by vertebrates in response to the development of strongly seasonal moisture variations associated with global climate change during the Permian and Triassic (e.g. [16]–[20]). In addition, this climate resulted in alkaline early diagenetic conditions that preserved both bone hydroxiapatite and coeval trace fossils due to the early cementation of the burrows by calcite cement [27].

In terms of paleoenvironmental evidence for the origin and preservation of the observed large-diameter burrows, the stratigraphic intervals in which the burrows are observed have common sedimentological similarities. From a sedimentological standpoint, both the Cancha de Bochas and Los Colorados burrows were formed in well-drained, overbank deposits of high-sinuosity fluvial systems. During the time of burrow development, the rates of lateral fluvial channel migration and floodplain aggradation were relatively low as evidenced by the diverse and apparently temporally-condensed accumulation of vertebrate fossils, as well as the well-developed paleosol morphologies that characterize the interval [20], [29]. However, periodic overbank deposition and overall positive accommodation development in the basin facilitated the burial and preservation of not only fossil material, but also the large-diameter burrows and individual paleosol horizons. Collectively, the depositional setting of both the Cancha de Bochas and Los Colorados burrowed intervals was likely in a sedimentologically-optimized habitat for the burrowing organisms. Specifically, the areas inhabited by the burrowers were far enough removed from fluvial channels to avoid seasonally elevated water tables or flooding events, but proximal to riparian environments to take advantage of surface or groundwater sources during water-stressed periods.

### Identifying the Possible Burrowing Organisms

Many different morphological characteristics have been utilized to identify potential architects of continental burrows including overall burrow architecture, superficial markings, dimensions, spatial relationships, and resemblance to known tetrapod burrows [4], [5], [71], [73]. The architectural and superficial morphologies described for Morphotypes 1 and 2 above indicate that the burrows were likely constructed by tetrapods.

The Morphotype 1 was previously interpreted as a tetrapod burrow cast by Colombi et al. [7]. Our interpretation of Morphotype 2 being produced by burrowing tetrapods as opposed to another potential producer of vertical/horizontal structures (e.g. crawfish, lungfish, or plant roots) is based primarily on the observed morphology of the structures. The large and relatively uniform diameter (∼30 cm) differs from that of crayfish burrows, whose maximum reported diameter is ∼8 cm (e.g. [4], [66], [77]). In addition, the architecture of Morphotype 2, characterized by in general more than one openings to the surface connected to undulatory horizontal tunnels and common enlargements associated with two or more convergent segments, differs significantly from the usually more complicated architecture of crayfish burrows or the bottle-like morphology of lungfish burrows (e.g. [66], [77]). The elliptical transverse section of Morphotype 2 is also unlike crayfish and lungfish burrows that have relatively circular cross-sectional geometries (e.g. [4], [66]). On the other hand, the well-defined contact between the coarser/calcite cemented sandstone burrow fill and the host lithologies are more consistent with a later filling of an empty burrow than a gradual filling of the space left by the progressive contraction of plant roots in the process of putrefaction as it is usually observed today. The way that tree trunks and woody roots are preserved in the Ischigualasto and Los Colorados Formations is always by the gradual replacement of the original organic structure by mineralizing fluids (silica in general) before the entire decomposition, as is possible to observe based on the preserved micro-structure [28]. Finally, neither those tree trunks nor roots show this type of undulatory feature accompanied by changes in the diameter (Figure 9c).

Besides the architecture, the only other parameter of these burrows that can be used to evaluate the possible burrower is the size. Burrow diameter is typically comparable to the body diameter of the animal that made it, so it is often possible to identify the burrower animal by the size of the burrow entrance [78], [79].

The burrows of Morphotype 1 in the Cancha de Bochas Member of Ischigualasto Formation have an average diameter of 10 cm, with an approximately elliptical cross-section. This diameter allows fossil vertebrates from the Ischigualasto Formation with a skull width or hip height greater than 10 cm to be disregarded as the potential burrower. This limit discards the majority of the known fauna from the formation, including the herrerasaurid dinosaurs, all crurotarsan archosaurs, rhynchosaurs, amphibians, dicynodonts, and traversodontid cynodonts. Similarly, the small dinosaurs (*Pisanosaurus*, *Eoraptor*, *Panphagia*, *Eodromaeus*, and *Chromogisaurus*), with an average hip height greater than 30 cm, are still bigger than the required size.

The only remaining burrower candidates are some of the faunivorous cynodonts identified in the Ischigualasto Formation (i.e. *Ecteninion*, *Probelesodon*, and cf. *Probainognathus*).

The burrows of Morphotype 2 of the Los Colorados Formation, with an average diameter of 30 cm, are larger than those of Morphotype 1 from the Ischigualasto Formation. Based upon known fossils from Los Colorados Formation, the sauropodomorph and theropod dinosaurs, dicynodonts, chelonians, and rauisuchids were all larger than this limiting diameter. The small to medium size aetosaurs, sphenosuchids, protosuchids, ornithosuchids, and cynodonts could be candidates as the producers and/or occupiers of the burrows. This long list of possible burrowers makes the determination of an individual candidate problematic. Because a minimal burrow diameter is thought to be key in reducing the energetic cost of excavation, the size of the burrow is closely related to the size of the producer [80]. As such, the smaller cynodonts *Chaliminia* and cf. *Tritylodon* and the protosuchid *Hemiprotosuchus* from the Los Colorados Formation seem to be too small (skull width of 2–3 cm) to be the burrow constructors. Other candidates, such as the ornithosuchid *Riojasuchus*, the aetosaur *Neoaetosauroides*, and the sphenosuchid *Pseudhesperosuchus*, are slightly bigger than the average burrow diameter. However, as previously noted, this does not exclude the possibility that they used the burrows during early ontogenetic stages.

Alternatively, two of the possible candidates for the architects of the Los Colorados burrows mentioned above, the cynodonts and aetosaurs, have antecedents with possible burrowing habits. The strongest evidence supporting the cynodonts as the burrowers is the discovery of several individuals of *Thrinaxodon* and *Trirachodon* in ancient burrows [8], [70], [71]. In addition, possible fossorial adaptations have been documented in the humerus of the cynodont *Irajatherium* [81], which is closely related to *Chaliminia*. As for the remaining group of possible burrowers, armored aetosaurs have been identified as potential constructors because some authors have speculated that the relatively massive limbs of aetosaurs, and especially the hypertrophy of muscular trochanters, suggest enhanced muscle power related to predominantly burrowing behaviors [49], [82]–[85].

Although enigmatic, Morphotype 3 has been interpreted as representing composite biogenic structures developed as a result of combined plant/animal interactions. Similar structures observed in Pleistocene deposits (1.5 Ma) in east Africa have been interpreted as calcified plant roots (network of long constant-diameter branches) modified by animal burrow chambers (elliptical or amorphous micritic-sandy masses amalgamated over the branches that give a chaotic aspect to the general structure) (A. K. Behrensmeyer personal communication, 2011). As such, Morphotype 3 may represent a possible early Mesozoic example of mutualistic plant-animal interaction preserved in the fossil record.

### Conclusions

Three ichnofossil morphotypes have been identified in Upper Triassic strata of the Ischigualasto-Villa Union Basin in northwestern Argentina. The first two are interpreted as tetrapod burrow casts, while the third has been interpreted as a composite form that developed as a result of mutualistic interactions between burrowing invertebrates and coeval root systems. In spite of the widespread outcrops of Upper Triassic rocks in South America, these morphotypes are the first to be studied in detail. One significant aspect of the observed structures is their association with floodplain facies that display evidence of seasonal and xeric conditions. These associations are in accord with interpretations of other Permo-Triassic burrows reported at different paleolatitudes of Pangea that suggest advanced burrowing behaviors were a mechanism to combat adverse climatic conditions. In addition, based on sedimentological interpretations, it is possible to conclude that the architect of the observed burrows selected an optimum environment far enough removed from fluvial channels to avoid seasonally elevated water tables or flooding events, but proximal to riparian environments to take advantage of surface or groundwater sources during water-stressed periods. Finally, we cannot identify with absolute certainty the architects of the burrows for either Morphotype 1 or Morphotype 2. However, for Morphotype 1, the strongest candidates are the small cynodonts *Probelesodon,* cf. *Probainognathus* and *Ecteninion*. These organisms were about the right size and have fossorial antecedents as a group, making them good candidates as the burrow architect. Unfortunately, for Morphotype 2, no known vertebrate from the relevant stratigraphic horizons has the appropriate size. The cynodonts are too small, and the small to medium-size archosaurs, at least in the adult stage, are somewhat larger than burrow diameters. It cannot be ruled out, however, that the observed burrows were occupied by the latter group in early ontogenetic stages.
